# Culture sensitive care in oncology care settings in Jordan: a qualitative study

**DOI:** 10.3389/fpubh.2026.1747462

**Published:** 2026-02-16

**Authors:** Omar Shamieh, Ghadeer Alarjeh, Waleed Alrjoub, Adib Edilbi, Ruba Al-Ani, Mousa Abdal-Rahman, Asem Mansour

**Affiliations:** 1 Department of Palliative Care, King Hussein Cancer Center, Amman, Jordan; 2 Center for Palliative & Cancer Care in Conflict (CPCCC), King Hussein Cancer Centre, Amman, Jordan; 3 School of Medicine, University of Jordan, Amman, Jordan; 4 Office of Director General, King Hussein Cancer Center, Amman, Jordan

**Keywords:** commination, culture, oncology, palliative care, sensitivity

## Abstract

**Introduction:**

Cultural beliefs significantly impact cancer perceptions, treatment choices, and care experiences. In Jordan, diverse patient background requires cultural competence and bias awareness among providers. This study aims to explore the cultural beliefs and practices of patients and their families in the experience of cancer care, and how these practices influence such care in Jordan.

**Materials and methods:**

This is a multi-center qualitative study used semi-structured face-to-face interviews with oncology healthcare providers, caregivers, and patients from three tertiary Jordanian hospitals providing cancer care. Purposive sampling was employed, and the interviews were analyzed using thematic analysis and NVivo 12 software.

**Results:**

We conducted 108 face-to-face interviews with 35 patients, 37 caregivers, and 36 providers. Notably, 22% of patients and caregivers were non-Jordanian. Thematic analysis identified five major themes shared across both Jordanian and non-Jordanian participants: (1) Tradition and Rituals: Preferences varied around food, visitations and modifications to usual religious practices, (2) Cancer Conceptualization: While many viewed cancer as a divine test, others attributed it to genetics, poor nutrition, stress, or environmental factors such as pollution, (3) Advance Directives and information sharing: Participants preferred timely discussions on directives with preferences ranging from proactive to gradual disclosure. Shared decision-making was emphasized. Typically involving physicians, family members, and key figures, especially the patient spouse or eldest male relative. Some caregivers and providers reported withholding details from patients and children upon family’s request, (4) Psychosocial and practical concerns: Participants expressed worries about treatment side effects, disease progression, financial burden, and the future of patient’ children, and (5) Healthcare Provider’s Characteristics: Patients preferred respect, and clinical expertise over providers’ gender, nationality, or religion. However they favored same-gender nurses for daily care and those who could communicate in Arabic.

**Discussion:**

The study demonstrates how culturally embedded beliefs, family dynamics, and faith-based interpretations uniquely shape cancer care interactions in Jordan. Addressing these context-specific cultural factors is essential for strengthening culturally responsive communication and patient-centered oncology services in the region.

## 1 Introduction

Culture encompasses shared values, beliefs, behaviors, and social structures that shape health perceptions, care-seeking behaviors, and interactions within healthcare systems (1). As a dynamic and socially constructed phenomenon, culture influences how illness is interpreted, how ethical uncertainties and moral distress arise, and how patients, families, and healthcare providers engage in decision-making processes (2, 3). In oncology care, these influences are particularly pronounced, as cancer trajectories often involve emotionally charged decisions, family negotiation, and end-of-life considerations.

To strengthen conceptual clarity, this study is informed by established cultural competence frameworks, including the Campinha-Bacote model, which emphasizes cultural awareness, knowledge, skill, encounters, and desire (4), and the Papadopoulos model, which conceptualizes cultural competence as a developmental process from awareness to culturally competent practice (5). Together, these frameworks provide an interpretive lens for understanding how culturally shaped interactions unfold within cancer care and guided the formulation of the research questions and analytic interpretation.

Healthcare systems increasingly serve culturally diverse populations, particularly in regions affected by migration and displacement. Migrant and refugee populations often present distinct health profiles and cultural expectations, posing challenges related to communication, decision-making, and institutional norms (6, 7). Culturally sensitive care defined as care that respects individuals’ values, beliefs, and social contexts has therefore become central to discussions of health equity, human rights, and social justice (8). Cultural factors also shape illness interpretation, perceptions of quality of life, expressions of pain, family roles, and engagement with health-related rituals, all of which complicate cancer care delivery (9, 10).

Jordan has experienced substantial population movement due to regional conflicts, intensifying cultural diversity within its healthcare system. While prior studies in Jordan and the broader Middle East have documented the influence of cultural beliefs on treatment decisions, pain management, and adherence to medical recommendations (11, 12), this evidence has largely been generated through quantitative designs or from healthcare provider perspectives. Consequently, limited attention has been paid to how patients and families themselves experience and navigate culturally embedded cancer care. Persistent cancer-related stigma, religious interpretations of illness, and misconceptions about treatment such as beliefs that surgery may spread cancer continue to influence care-seeking behaviors and trust in medical services (13).

Cultural experiences of cancer care are inherently relational, value-laden, and embedded within social and family contexts. These experiences shape how diagnoses are communicated, how decisions are negotiated, and how care is ultimately received, yet they cannot be adequately captured through quantitative measures alone. A qualitative approach is therefore essential to explore the meanings patients and families assign to cancer care encounters, particularly in contexts where cultural norms strongly influence communication, shared decision-making, and expectations of care (14–16).

Despite growing recognition of the importance of culturally responsive oncology practice in the Middle East, qualitative evidence examining culturally grounded cancer care experiences remains limited in Jordan. Existing research has focused predominantly on clinical outcomes or provider-reported perspectives, leaving a critical gap in understanding cancer care trajectories from the viewpoints of patients and caregivers. Building on calls for patient-centered cancer care (17) and drawing on experiences at the King Hussein Cancer Center, this study employs qualitative inquiry to explore how cultural beliefs and practices shape cancer care in Jordan. By foregrounding patient, caregiver, and healthcare provider perspectives, the study aims to generate context-sensitive insights that can inform culturally responsive care models, enhance communication and shared decision-making, and ultimately improve the quality and equity of oncology care.

## 2 Materials and methods

### 2.1 Design

Our study employed a multi-center qualitative research design that used semi-structured, audio-recorded interviews to explore the experiences, beliefs, and perspectives of patients with cancer, caregivers and healthcare providers (HCPs), We collected data on cultural beliefs related to social structures, traditions, rituals, and other factors that might have influenced patient treatment trajectories, decision-making preferences, and overall satisfaction. This study adopted a qualitative design using reflexive thematic analysis informed by an interpretivist paradigm. This approach was selected to identify and interpret patterns of meaning across participants’ narratives, with particular attention to how cultural values and social norms shaped experiences of cancer care. The analytic aim was not theory generation but rather an in-depth understanding of shared and divergent experiences within a specific sociocultural context (18).

### 2.2 Sampling

Participants were recruited using purposive sampling to capture a range of perspectives relevant to culturally sensitive cancer care. Dimensions of variation included age, gender, cancer type, disease stage, and family caregiving role. Eligible individuals were identified through oncology clinics and inpatient departments across the three participating institutions and were then contacted via telephone by the clinical team to invite them to participate. Sample size was determined using the principle of data saturation. Data collection and analysis were conducted concurrently, allowing emerging themes to be continuously assessed across interviews. Interviews continued until no substantively new themes or insights were identified in successive interviews. Regular analytic discussions within the research team were used to evaluate thematic repetition and confirm that additional interviews were unlikely to yield novel or meaningful information. Data collection was therefore discontinued once saturation was achieved, ensuring sufficient depth and conceptual completeness of the findings (19).

### 2.3 Participants and settings

We included three stakeholders groups: adult patients (above 18 years) with cancer, their adult caregivers and healthcare providers with at least 1 year of experience in cancer care. Participants were recruited from three hospitals: King Hussein Cancer Center (KHCC), King Abdullah University Hospital (KAUH), and Al Basheer Hospital.

The three study sites were selected to capture diverse perspectives, as each hospitals treat Jordanian and non-Jordanian patients including refugees. The King Hussein Cancer Center (KHCC), a leading a non-governmental tertiary cancer center that treat more that 60% of cancers case from Jordan and surrounding region (10). Albasheer Hospital, the largest public hospital in the country delivers care across a wide range of diseases, including cancer to both Jordanian and non-Jordanian patients. Both KHCC and Albasheer hospital are located at the capital city of Jordan, Amman. While KAUH is a university hospital that treat patients and refugees in the north of Jordan in Irbid city for different kind of diseases including cancer. Together, these institutions represent the breadth of cancer care delivery across private, public, and academic sectors in Jordan.

### 2.4 Collecting data and ethical consideration

#### 2.4.1 Data collection procedures

Between June 2020 and May 2021, semi-structured interviews were conducted in Arabic by four trained research assistants (GA, WA, RA, and AE), each with prior experience in qualitative interviewing. Recruitment began with the research assistants liaising with medical and nursing staff across outpatient and inpatient departments to identify eligible participants. In addition, clinic and hospital admission lists were reviewed to further ensure comprehensive identification of potential participants.

Eligible individuals were then contacted via telephone across the three participating institutions. During the initial contact, detailed information regarding the study’s aims, procedures, and ethical considerations was provided. Participants were given sufficient time to consider their involvement. Informed consent was obtained either in writing or verbally for participants interviewed virtually by phone. For those recruited by phone, verbal consent was audio-recorded following participant approval.

Ethical approval for the study was obtained from the Institutional Review Board of King Hussein Cancer Center (Reference: 19 KHCC 106). The other participating sites provided formal agreement letters recognizing the KHCC IRB approval.

### 2.5 Guide development and interview procedure

Semi-structured interview guides were developed for healthcare providers, patients, and caregivers, drawing on the literature and expert input. Guides were pilot-tested and iteratively refined based on early interviews and team reflection to ensure clarity, relevance, and sensitivity. Interviews were conducted by trained qualitative researchers, either in person or by telephone according to participant preference and health status. Telephone interviews followed the same guide and probing strategies, with adaptations to maintain rapport and narrative depth despite limitations in observing non-verbal cues. Interviewers were trained to manage emotional distress, allowing participants to pause, redirect, or terminate discussions as needed, and referral support was available when required. Patients could include a caregiver, whereas caregivers and healthcare providers were interviewed individually to preserve privacy and minimize response bias. All interviews were audio-recorded, transcribed verbatim in Arabic, and anonymized. Field notes were recorded after each session to capture contextual details, non-verbal cues, and reflexive observations. Interview duration was adapted to participant stamina and cultural norms, particularly for patients with advanced illness, balancing fatigue with the need for rich, in-depth narratives.

### 2.6 Reflexivity

All researchers engaged in ongoing reflexive practices throughout data collection and analysis. While no team member had personal migration experience, interviewers shared cultural and linguistic familiarity with participants, facilitating rapport while requiring attention to avoid assumptions. Reflexive memos and team discussions were used to critically examine how researchers’ social, cultural, and professional positioning might influence interpretation, ensuring that the analysis remained grounded in participants’ narratives (20)

### 2.7 Data analysis

Participant characteristics were analyzed descriptively by GA and MA, as presented in Table 1. Interviews were transcribed verbatim by RA and AE. All transcripts were pseudonymized to ensure confidentiality and subsequently imported into NVivo 12 Pro for data management and analysis.

**Table 1:** Characteristics of the study participants (*N* = 108).

Category	Patients (*n* = 35)		Caregivers (*n* = 37)		HCPs (*n* = 36)	
	*N*	%	*N*	%	*N*	%
Gender						
Female	18	51	23	62	18	50
Male	17	49	14	38	18	50
Nationality						
Jordanian	29	83	27	73	36	100
Libyan	2	6	1	3	—	—
Palestinian	3	9	4	11	—	—
Syrian	1	2	5	13	—	—
Cancer diagnosis						
GI	11	34	—	—	—	—
Breast	6	17	—	—	—	—
Lung	6	17	—	—	—	—
Renal tumors	2	5	—	—	—	—
Gynecological	3	9	—	—	—	—
Hematological	3	9	—	—	—	—
Sarcomas	3	9	—	—	—	—
Cancer stage						
Stage IV	21	60	—	—	—	—
Stage III	8	23	—	—	—	—
Stage II	5	14	—	—	—	—
Stage I	1	3	—	—	—	—
Caregiver relationship						
Spouse	—	—	12	32	—	—
Daughter	—	—	7	20	—	—
Son	—	—	6	16	—	—
Parent			6	16	—	—
Sibling			6	16	—	—
Professional role						
Physicians	—	—	—	—	13	36
Nurses	—	—	—	—	21	58
Psychosocial	—	—	—	—	2	6
Marital status						
Married	28	80	26	70	25	69
Widow/Widower	4	11	1	3	—	—
Divorced	2	6	1	3	1	3
Single	1	3	9	24	10	28
Has children						
Yes	33	94	24	65	25	69
No	2	6	13	35	11	31
Education						
BSc	14	40	13	36	36	100
High School	10	29	12	32	—	—
Preliminary school	11	31	12	32	—	—

HCPs = Healthcare Professionals; GI = Gastrointestinal; BSc = Bachelor’s.

Although each guide was tailored to the participant group, all were structured around shared overarching domains including cultural beliefs and rituals, conceptualization of illness, communication and decision-making preferences, psychosocial and practical concerns, and perspectives on healthcare providers. These parallel domains enabled direct comparison across groups.

Analytically, we applied a unified thematic framework to all transcripts, allowing cross-stakeholder triangulation. The research team compared how each group described similar cultural phenomena, examining convergences and divergences to enhance rigor and depth. This approach ensured that variations in guide wording did not hinder thematic consistency or comparability. We also clarified these procedures in the revised manuscript.

Thematic analysis was conducted using Braun and Clarke’s six-phase framework for both inductive and deductive coding (Table 2) (21). GA and WA led the coding process, with initial theme development followed by multiple rounds of team discussions to refine the thematic framework. This iterative process involved reorganizing, adding, modifying, and eliminating codes and themes to ensure conceptual clarity and analytical rigor. Once consensus was achieved among all team members regarding the thematic structure and interpretation, GA and WA applied the finalized framework across the entire dataset.

**Table 2:** Phases of thematic analysis process.

Phase	Description
Phase 1: Immersion in data	Interview transcripts were repeatedly read for deep understanding. Audio recordings were transcribed verbatim by RA and AE and revised accuracy by GA and WA to ensure familiarity with content.
Phase 2: Initial coding	Preliminary codes were independently generated by the research team (GA and WA). Coding was refined through regular face to face meetings to reach consensus.
Phase 3: Theme development	Codes were organized into potential themes. GA drafted the preliminary thematic framework.
Phase 4: Theme refinement	Themes were collaboratively reviewed for clarity and coherence. Redundant themes were removed, merged, or subdivided based on content differentiation.
Phase 5: Defining themes	Themes were refined and clearly labeled to reflect their conceptual scope, ensuring alignment with the overall dataset.
Phase 6: Report production	The final analysis was documented through an interpretative narrative supported by illustrative quotes drawn directly from participant interviews and then translated into English.

The analysis was conducted in Arabic to maintain linguistic and contextual accuracy. Final themes and quotations were translated into English by two bilingual translators, then back translated into Arabic by two others. To ensure translation reliability, two research team members (GA and MA) conducted a comprehensive quality assurance review, confirming consistency and accuracy across all translated content.

## 3 Results

### 3.1 Characteristics of participants

A total of 108 semi-structured interviews were conducted across three hospitals in Jordan, with an equal distribution of 36 interviews per site; 30 interviews (27.8%) were conducted via phone. Of the total sample, 92 participants (85%) were Jordanian nationals and about 22% of patients and caregivers were non-Jordanian. Participation rates were high, with approximately 90% of those approached consenting to take part; refusals were primarily due to time constraints, fatigue, or lack of interest.

The median interview duration was 36.5 min (range: 25–59). By group, median durations were healthcare providers (41 min), patients (37.6 min), and caregivers (31.5 min). Of the 35 patients interviewed, 20 (57%) were accompanied by their caregivers during the interview. Patients had a mean age of 52.97 years (SD = 10.45), caregivers 40.41 years (SD = 13.16), and healthcare providers 34.4 years (SD = 7.35), with providers reporting a mean of 9.03 years of experience (SD = 5.02). Gender distribution was balanced among patients and HCPs, while 62% of caregivers were female. Most participants were Jordanian. Among patients, gastrointestinal, breast, and lung cancers were the most common diagnoses, with 83% presenting at advanced stages. Primary caregivers were most often spouses or daughters. Educational backgrounds varied, with many holding high school or bachelor’s degrees. Most healthcare providers were nurses or physicians (Table 1).

### 3.2 Main themes

**Figure 1:**
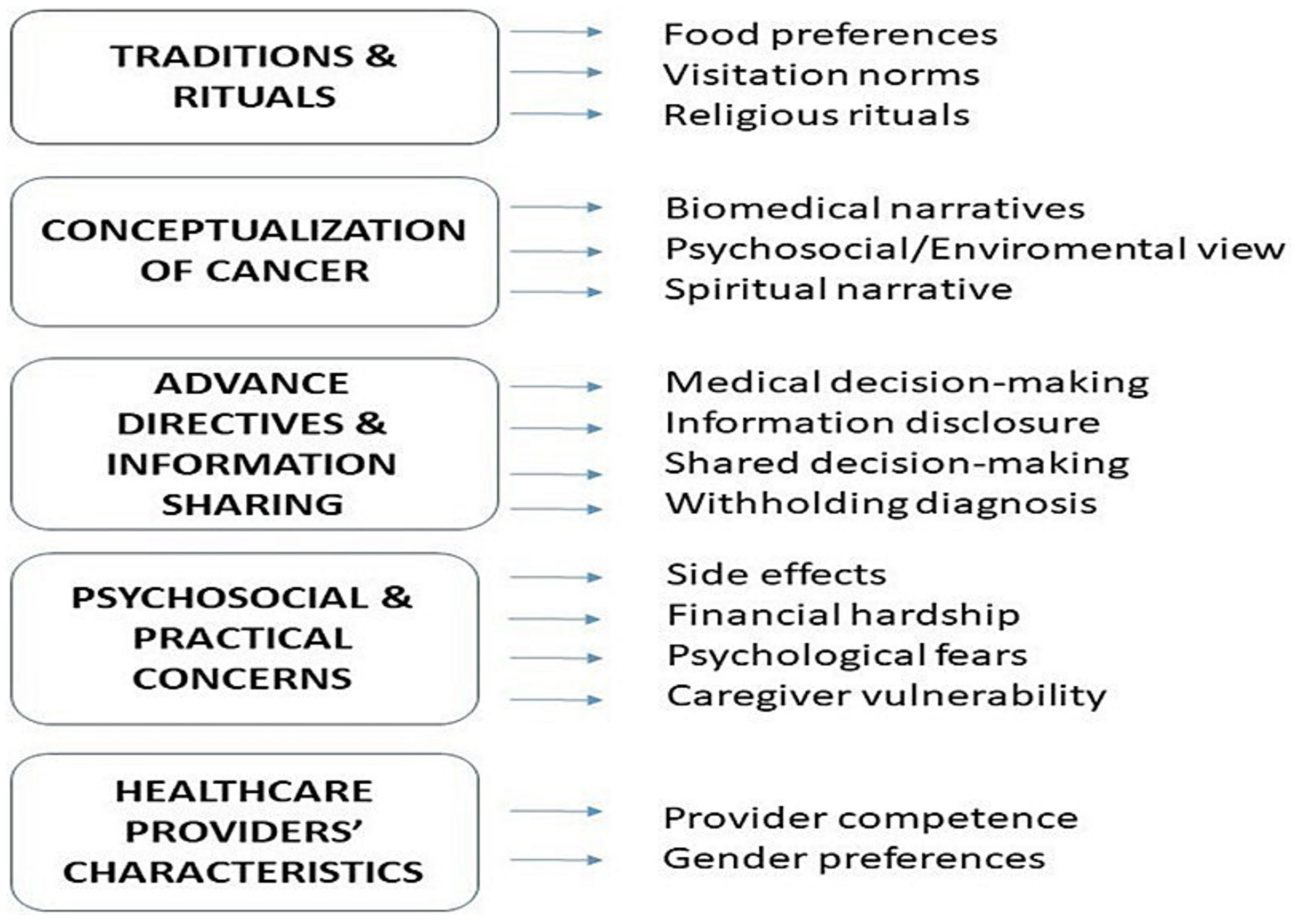
Thematic map of 5 major themes and subthemes identified from qualitative interviews with oncology patients, caregivers, and healthcare providers at 3 sites in Jordan.

The thematic analysis revealed five interrelated themes reflecting the lived experiences, cultural values, and psychosocial concerns of cancer patients and their caregivers: (1) Tradition and Rituals, (2) Conceptualization of Cancer, (3) Advance Directives and Information Sharing, (4) Psychosocial and Practical Concerns, and (5) Healthcare Providers’ Characteristics (Figure 1). These themes illustrate the intersection of cultural beliefs, personal values, and healthcare experiences across a diverse patient and caregiver population.

#### 3.2.1 Traditions and rituals

Across patient and caregiver interviews, participants described how deeply embedded cultural beliefs and everyday practices particularly those related to food preferences, visitation norms, and religious rituals played a central role in shaping their hospital experiences and care-related decisions. Several patients and caregivers highlighted food practices as central to maintaining familiarity and continuity during illness. Hospital-provided meals were often described as misaligned with customary diets, prompting reliance on homemade food. Participants’ accounts illustrate how these practices influenced comfort, meal acceptance, and perceived quality of care.

*“Honestly, look, there’s food in the hospital that I completely refused, and I used to bring food from outside. I mean, it’s rice every day, if you keep eating the same thing for 2 or 3 days, you start to really hate it. You have to have* var*iety. So in this hospital, I always returned the rice; I never took it.”* (75 years old male patient, Palestinian)

*“The food in the hospital was very different from what we eat at home. I could not eat most of it, so my family started bringing food from home. When I ate our own food, I felt more comfortable and a little more like myself, even though I was sick.”* (41 years old male patient, Jordanian)

Participants consistently emphasized the emotional importance of hospital visits, while also describing the physical and psychological burden of excessive visitation. Preferences varied depending on patients’ physical condition and psychological needs. Some participants favored short, scheduled visits from close, emotionally supportive individuals while discouraging visits from young children or visibly distressed relatives. Visits were seen as emotionally uplifting yet potentially exhausting if not carefully managed.

*“Honestly, it’s not always that a person likes visits. Sometimes visits can exhaust a person. Too many visits can be tiring. There’s a specific time for visits in the hospital, and that’s when a person can receive people. But more than that, the patient might get exhausted. But one must visit others, it’s important. Visits sometimes give encouragement to the patient. Some people uplift the patient, give them morale, a lot of morale, more than if they did not see anyone.”* (59 years old male patient, Jordanian)

*“No, children, better not. The impact on their psyche would be bad, first of all. And besides that, their immunity is weak, they could catch diseases. No, I prefer children not to come.”* (53 years old male caregiver, Jordanian)

Among both patients and caregivers, religious rituals emerged as a prominent coping strategy. Participants repeatedly described reliance on prayer, Qur’an recitation, spiritual healing, and charitable acts as sources of solace and resilience, as illustrated in the quotations presented.

*“Supplications and the Qur’an give you a spiritual boost. In the end, God is the only one who has power. A person turns to the Lord of the Worlds… I used to read the Qur’an and make supplications, but now I do more, I supplicate more and read more Qur’an.”* (59 years old male patient, Jordanian)

#### 3.2.2 Conceptualization of cancer

Participants articulated diverse explanatory models of cancer, including biomedical, environmental, psychosocial, and spiritual interpretations. These explanatory frameworks were evident across interviews and shaped expectations, coping strategies, and treatment-related decisions, as demonstrated in participants’ narratives.

*“there is a genetic factor, yes. And she did experience a shock, honestly. And no doubt, the nutritional factor plays a main role. there are some bad things. Like hormone-injected food …. In our household we completely cut out meat, because this illness feeds on red meat, and sugars.”* (35 years old male caregiver, Syrian)

For many participants, cancer was ultimately framed through a spiritual lens, described as a divine test, a means of expiation, or a trial of faith. A smaller number of participants mentioned alternative interpretations, such as the evil eye or environmental conspiracies, underscoring the varied ways participants sought meaning in illness.

*“In the end, I believe my health and my illness are in God’s hands. Whether it came from stress, genetics, or pollution doesn’t really matter, God is the one who chooses our tests. This faith is what gives me the strength to accept the disease and continue with the treatment.”* (47 year old female patient, Jordanian)

*“I mostly see that cancer patients have psychological and social problems, and also genetic issues. Social conditions like poverty or trauma play a role. Many patients from refugee camps say it’s from the war, from the chemicals, from the life they’re living.”* (50 years old female, nurse)

*“At the beginning, some of us believed that what happened might be because of the evil eye. Others in the family also talked about things in the environment, like pollution or chemicals, as possible reasons. We were trying to understand why this illness happened to him.”* (47 years old female caregiver, Jordanian)

#### 3.2.3 Advance directives and information sharing

Patients and caregivers’ accounts revealed a spectrum of preferences regarding information disclosure and medical decision-making, ranging from passive acceptance to active engagement. These preferences were shaped by participants’ experiences, trust in healthcare providers, cultural norms, and perceived ability to process information.

*“I like to be informed, and I like to communicate with the doctor so that he explains to me step by step what is going to happen to me before he starts, and he tells me that there’s step one, two, three, four, so we can follow them. He asks my opinion, and I review them for an hour or two and agree to them if I think they’ll benefit me.”* (75 years old male patient, Palestinian)

*“it’s okay if he gives me the plan within a slightly broader scope than just the narrow one we’re currently in, not only “if this happens, we’ll do that.” Maybe, yes, but not too much expansion. Stages are better. I mean, he gives me a general overview, a complete general view, not detailed, and gives me detailed information for each stage.”* (53 years old male caregiver)

*“Some of the time, I preferred that the doctor just tell us what to do, because I trusted their experience and didn’t feel able to understand everything. Other times, especially when decisions were serious, I wanted to ask questions and be involved. It depended on how confident I felt and how much I could handle at that moment.”* (40 years old male caregiver)

Several participants advocated for shared decision-making involving physicians, family members, particularly spouses or male relatives and patients. Cultural norms and religious beliefs frequently guided who was included in these decisions. Educated families were more likely to favor collaborative approaches, while others deferred entirely to physicians or senior family members.

*“I prefer shared decision-making because sometimes the patient might be unaware of certain things, and someone from my relatives could point something out to me, but the first and final say goes back to the patient. I mean, with all due respect, My father, the head of the household, just like I’m responsible for him, he’d be responsible for me. In a family, as they say, we carry each other.* (26 years old male caregiver, Jordanian)

*“In our family, we believe decisions should be made together with the doctor and close family members. Because we are educated, we like to discuss the options, ask questions, and understand the plan. At the same time, our cultural and religious values emphasize respect for elder relatives. Other families may prefer to leave decisions completely to the doctor or to the eldest family member.”* (20 years old male caregiver, Jordanian)

Some caregivers reported intentionally withholding diagnostic information from patients, particularly children and older adults, with the stated aim of protecting them from emotional distress. Healthcare providers’ accounts corroborated these practices, reinforcing the consistency of this theme across data sources.

*“Some children grow up without knowing they have cancer, as families often say it’s a routine illness to avoid scaring or burdening them. Adults want to protect their mental well-being, but it can also be due to not knowing how to explain cancer or its treatment. Society still views cancer as frightening or serious, which can influence families to hide the diagnosis.”* (25 years old female, nurse)

*“We didn’t tell my mother the full details about her diagnosis because we were afraid it would upset her. We just wanted to protect her from worrying too much.”* (46 years old male caregiver, Jordanian)

Conversely, some participants described themselves as fully autonomous, insisting on making medical decisions independently and advocating for full disclosure even if the news was distressing.

*“The person who makes the decision should be the one who has the health issue, the one experiencing the problem, you get what I mean? First, we discuss, like, what the solution is and what we want to do. And then, after that, it’s basically up to you. You’re the one with the health issue, the one suffering from it.”* (36 years old female caregiver, Jordanian)

#### 3.2.4 Psychosocial and practical concerns

Participants described a broad range of physical, psychological, and existential concerns that recurred across interviews. Treatment side effects, financial strain, fear of disease progression, and loss of independence were frequently raised, highlighting the multifaceted burden of cancer.

*“The thing they fear the most is that the illness will weaken them, that they’ll lose their hair, this is something that really matters to them. For example, with males and females, especially young people, they worry, “Will I be able to have children or not?” Things like pain, feeling broken, becoming more vulnerable to other diseases these are all concerns. The side effects are a big deal.* (33 years old female, doctor)

Many participants addressed the financial hardship emerged as a major source of anxiety, especially among patients who were the family’s primary earners. Some worried about affording long-term treatments, while others contemplated the cost of their own burial, underscoring the deep emotional and practical burden of cancer.

*“the patient starts having internal conflicts like, for sure there’s a part of them deep down saying, “I need to get closer to God; my days are numbered. I feel like I could die at any moment I have cancer, I’m sick.” Then they start thinking about their kids, “Where will my children go? Who’s going to take care of them? Who’s going to support them financially?” Especially if he’s the sole breadwinner. Some even start thinking, “Where will they get the money for my shroud? How will I be buried? Who will handle the funeral?” Imagine, they are thinking about the things that will happen after they die.* (25 years old female, nurse)

Psychological fears centered on loss of independence, becoming a burden to others, and facing mortality. The association of palliative care with death often heightened patients’ anxiety at initial referral, though perceptions tended to shift positively after receiving supportive care.

*“Honestly, auntie, I just ask that my health stays good and stable, and that I don’t need anyone. This medicine I take every 21 days, I’ll be on it for life, as long as I’m alive, you know? I pray to God that I can keep taking the medicine and walk on my own two feet, not needing anyone. That’s all I wish for, nothing more. Thank God.”* (55 years old female patient, Jordanian)

*“Many patients initially fear palliative care because they see it as the end, as losing control over their lives, or being a burden to their families. But after they start receiving support, I often see their anxiety lessen and their outlook become more positive.”* (33 years old female, doctor)

Caregivers, particularly sons and daughters, expressed intense emotional vulnerability linked to the anticipated loss of parental figures, who were often seen as their moral and emotional anchors. These sentiments were especially pronounced among younger caregivers and those from close-knit families.

*“My biggest fear is losing my father. He’s my support system, like my right hand. He’s the advice, the guidance. Losing him would change everything.”* (26 years old male caregiver, Jordanian)

#### 3.2.5 Healthcare providers’ characteristics

While professional competence and communication skills were consistently prioritized across interviews, participants described gender preferences in specific clinical contexts. In particular, preferences for same-gender providers were expressed in relation to intimate nursing care, reflecting cultural norms related to modesty.

*“Honestly, whether HCP were male or female, it depends on their knowledge. I prefer whoever has the expertise… but for daily care, I prefer a male nurse because we’re the same gender. With a female nurse, it’s difficult for me… I feel modest”* (32 years old male, Libyan)

*“Healthcare Provider: Well, regarding doctors, gender dose not matter, when it comes to nursing, there are usually more personal or intimate needs involved, you know what I mean, so naturally, a person feels more comfortable with someone of the same gender. But if it’s just doctors, the examinations are usually simple, so it doesn’t really matter as long as the doctor is competent and God-fearing.”* (44 female nurse, Jordanian)

Most participants reported no objection to providers’ religion or nationality, emphasizing that professional ethics and compassion were more important than identity. However, Arabic-speaking providers were preferred by some patients and caregivers due to ease of communication, cultural resonance, and reduced misunderstandings.

*“I have no problem with my health care provider nationality or religion however, I prefer, just prefer an Arab, a free Arab person. Honestly, if a foreign doctor comes, maybe I’ll understand a couple of words and not understand others. So, in Arabic, it’s better.”*(75 years old male patient, Palestinian)

## 4 Discussion

This multi-center qualitative study advances understanding of culturally embedded cancer care by demonstrating how beliefs and everyday practices in Jordan function as mechanisms that actively shape communication, decision-making, and care experiences, rather than serving as passive background characteristics. While prior studies have documented similar cultural dimensions in oncology and palliative care (22, 23), our findings are analytically distinctive in illustrating how these dimensions operate within a Jordanian sociocultural context characterized by strong family interdependence, faith-based meaning-making, and moral obligations surrounding protection and responsibility. One of the most salient contributions of this study is the role of traditions and rituals in preserving identity, comfort, and continuity during the cancer trajectory. Preferences for homemade food reflect what anthropological literature describes as symbolic nourishment, where food serves not only nutritional needs but also emotional and spiritual functions (24). In our data, hospital meals were often perceived as impersonal or culturally incongruent, disrupting patients’ sense of normalcy and prompting reliance on family-provided food. This finding aligns with Larsen et al.’s (2021) work highlighting how institutional food environments influence patients’ motivation to eat, yet extends it by showing how cultural meaning attached to food mediates engagement with hospital care in Jordan (25). Visitation practices and religious rituals similarly acted as stabilizing forces, offering emotional reassurance and reinforcing social bonds patterns consistent with evidence that spiritual and relational practices enhance quality of life in advanced cancer across settings, but here deeply embedded in collective cultural norms (26).

Participants’ conceptualizations of cancer further demonstrate how cultural beliefs shape care experiences through pluralistic explanatory models. While biomedical and environmental explanations such as genetics, pollution, war exposure, and diet were common, many participants simultaneously interpreted cancer as a divine test or spiritually meaningful experience. These interpretations influenced coping strategies, treatment acceptance, and communication with healthcare providers. Unlike findings from Australian and Vietnamese contexts, where cancer risk is often attributed primarily to aging or nutrition, participants in this study integrated spiritual, emotional, and sociopolitical explanations, underscoring how illness meaning is constructed within broader moral and historical contexts (27).

Decision-making and information-sharing practices revealed another analytically distinctive feature: the predominance of relational autonomy. Consistent with Yennurajalingam et al.’s (2018) international findings showing variability in decisional control preferences, our data illustrate that many Jordanian patients favored shared or family-mediated decision-making (28). Authority was often distributed among physicians, patients, and key family members, particularly spouses or senior male relatives reflecting cultural norms of collective responsibility. In this context, autonomy is not conceptualized as individual independence but as relational, negotiated, and embedded within family structures, aligning with broader ethical discussions on relational autonomy in clinical care (29).

Information withholding, particularly from children or older adult(s) patients, further highlights how protective intentions rooted in care and moral responsibility shape communication practices. Similar patterns have been observed in pediatric oncology settings in Jordan, suggesting a culturally consistent approach to disclosure aimed at minimizing psychological harm (7). While such practices may conflict with Western norms of full transparency, our findings emphasize the importance of culturally sensitive negotiation rather than prescriptive application of disclosure standards.

Psychosocial and practical concerns illuminated how cancer disrupts entire family systems, not only individual patients. Fears of disease progression, physical decline, and becoming a burden echoed findings from palliative care studies in other contexts (30, 31), yet in Jordan these concerns were deeply intertwined with cultural expectations regarding breadwinning, caregiving, and intergenerational responsibility. Patients’ anxieties about their children’s futures and caregivers’ fears of losing moral and emotional anchors underscore the relational nature of suffering and highlight the need for family-centered supportive interventions.

Preferences regarding healthcare providers’ characteristics further reinforce the importance of cultural congruence in care delivery. While clinical competence was universally prioritized, preferences for same-gender nurses during intimate care and for Arabic-speaking providers reflected values of modesty, privacy, and trust. These findings parallel evidence from Saudi Arabia emphasizing the role of cultural alignment in nursing care, while underscoring practical implications for staffing, training, and communication strategies in culturally diverse oncology settings (32).

Importantly, the inclusion of non-Jordanian participants many from migrant or displaced backgrounds situates these findings within broader regional and global dynamics of migration and conflict (10). The mechanisms identified here are therefore relevant not only to Jordan but also to other healthcare systems serving Arab, Muslim, and collectivist populations. By clarifying how beliefs and practices shape care experiences, this study provides empirically grounded insights to inform culturally responsive communication, shared (relational) decision-making, and service adaptation for diverse and migrant populations.

Collectively, these findings extend existing literature by moving beyond descriptive accounts of cultural beliefs to demonstrate how culture actively structures cancer care experiences. Integrating such insights into clinical training and institutional policies is essential for advancing cultural competence as a core component of equitable, patient-centered oncology care.

### 4.1 Strengths and limitations

This study has many notable strengths. It represents one of the first multi-center qualitative research studies in Jordan to examine how cultural beliefs shape cancer care, offering rich, context-specific insights. By involving patients, caregivers, and healthcare providers, the research captured variety of perspectives and provided a more holistic understanding of cultural influences on treatment and care delivery. The diverse sample across three hospitals enhanced the transferability and richness of the findings. Furthermore, conducting interviews in Arabic preserved cultural nuance during data collection, while the use of thematic analysis supported by NVivo software ensured a systematic and rigorous approach to interpretation. Importantly, the study’s findings have direct implications for both practice and policy, highlighting opportunities to improve culturally sensitive, patient-centered cancer care.

On the other hand, some limitations must be acknowledged. As with most qualitative research, the findings cannot be generalized to all patients with cancer in Jordan or to other contexts. Recruitment from tertiary hospitals may have excluded the perspectives of patients receiving care in rural or resource-limited settings. In addition, some interviews were conducted by phone during the COVID-19 pandemic, which may have limited rapport and the ability to capture non-verbal communication cues. Cultural sensitivity is also a dynamic and context-specific concept, meaning that the identified themes may change over time or differ in other cultural or clinical environments. Finally, because the study addressed sensitive issues such as disclosure, family dynamics, and religious practices, some responses may have been influenced by social desirability bias.

## 5 Conclusion

This study directly addresses how cultural beliefs and practices shape the cancer care journey in Jordan, influencing communication preferences, decision-making patterns, coping strategies, and perceptions of family responsibility. By clarifying these culturally grounded mechanisms, the study identifies opportunities for improving culturally responsive and patient-centered oncology care.

From food to faith, from communication preferences to caregiving dynamics, the findings reveal that healthcare is not merely a set of procedures, but a relational, spiritual, and emotional landscape traversed by patients and families alike.

Understanding how cultural values shape preferences for information sharing, spiritual coping, and interpersonal boundaries can enhance patient-centered care. Healthcare providers must move beyond biomedical checklists and engage with the patient’s worldview, recognizing that illness is as much a social and moral disruption as it is a physical one.

Just as a sailor reads not only the map but also the wind, healthcare teams must attune themselves not only to clinical data but also to cultural and emotional cues. This requires training in communication, empathy, and cultural humility, especially in regions with strong familial ties and spiritual belief systems.

Future research should explore how these themes vary across age, gender, and socio-economic strata and how healthcare systems can design culturally congruent models of care that are both ethically sound and practically feasible. Ultimately, the integration of cultural competence into cancer care is not an optional luxury but a fundamental pillar of equitable, compassionate, and effective medicine.

## Data Availability

The original contributions presented in the study are included in the article/supplementary material, further inquiries can be directed to the corresponding author/s.
